# Rate of Pathogenic Germline Variants in Patients With Lung Cancer

**DOI:** 10.1200/PO.23.00190

**Published:** 2023-11-22

**Authors:** Steven Sorscher, Jaclyn LoPiccolo, Brandie Heald, Elaine Chen, Sara L. Bristow, Scott T. Michalski, Sarah M. Nielsen, Alix Lacoste, Emil Keyder, Hayan Lee, Robert L. Nussbaum, Renato Martins, Edward D. Esplin

**Affiliations:** ^1^ Invitae, San Francisco, CA; ^2^ Hematology/Oncology Division, Dana-Farber Cancer Center, Boston, MA; ^3^ Nuclear Dynamics and Cancer Program, Cancer Epigenetics Institute, Fox Chase Cancer Center, Philadelphia, PA; ^4^ Hematology, Oncology and Palliative Care Division, Massey Cancer Center, Virginia Commonwealth University, Richmond, VA

## Abstract

**PURPOSE:**

Germline genetic testing (GGT) is now recommended for all patients diagnosed with ovarian or pancreatic cancer and for a large proportion of patients based solely on a diagnosis of colorectal or breast cancer. However, GGT is not yet recommended for all patients diagnosed with lung cancer (LC), primarily because of a lack of evidence that supports a significant frequency of identifying pathogenic germline variants (PGVs) in these patients. This study characterizes GGT results in a cohort of patients with LC.

**METHODS:**

We reviewed deidentified data for 7,788 patients with GGT (2015-2022). PGV frequencies were compared to a control cohort of unaffected individuals. GGT results were stratified by genomic ancestry, history of cancer, and PGV clinical actionability per current guidelines.

**RESULTS:**

Of all patients with LC, 14.9% (1,161/7,788) had PGVs. The rate was similar when restricted to patients with no cancer family history (FH) or personal history (PH) of other cancers (14.3%). PGVs were significantly enriched in *BRCA2*, *ATM*, *CHEK2*, *BRCA1*, and mismatch repair genes compared with controls. Patients of European (EUR) genomic ancestry had the highest PGV rate (18%) and variants of uncertain significance were significantly higher in patients of non-EUR genomic ancestry. Of the PGVs identified, 61.3% were in DNA damage repair (DDR) genes and 95% were clinically actionable.

**CONCLUSION:**

This retrospective study shows a LC diagnosis identifies patients with a significant likelihood of having a cancer-predisposing PGV across genomic ancestries. Enrichment of PGVs in DDR genes suggests that these PGVs may contribute to LC cancer predisposition. The frequency of PGVs among patients with LC did not differ significantly according to FH or PH of other cancers.

## Teaser

14.9% of patients with lung cancer were identified as carriers of a well-described pathogenic germline variant.

## INTRODUCTION

Lung cancer (LC) is the leading cause of cancer mortality worldwide. Pathogenic (P)/likely pathogenic (LP) germline variants (PGVs) appear to increase LC risk, given an estimated 18% LC risk associated with family history (FH).^1,2^ Reported PGV rates in patients with LC range from 0.3% to 7%.^3-10^ Studies of paired tumor-normal samples across multiple cancer types found PGV rates of 14.1% and 5.8% in patients with advanced LC.^11,12^ Samadder et al^13^ reported a similar PGV rate (14.7%) in an unselected LC cohort. Although *TP53* (Li-Fraumeni syndrome) and *EGFR* (p.T790M) PGVs clearly predispose to LC,^3,14-16^ most PGVs are not proven to be LC-predisposing.^13^

**CONTEXT**

**Key Objective**
Identify the frequency of pathogenic germline variants (PGVs) in a real-world setting in which the basis for germline genetic testing (GGT) was ostensibly a diagnosis of primary lung cancer (LC).
**Knowledge Generated**
The finding that 14.9% (1,161/7,888) of patients diagnosed with LC carry PGVs supports Cancer Moonshot 2.0's recommendation that nearly all patients with cancer be offered referral for genetic counseling. The frequency of particular DNA damage repair PGVs identified warrants further studies to establish whether these PGV-like EGFR p.T790 are LC-predisposing.
**Relevance**
Obtaining thorough and accurate personal and family histories and tumor genetic testing remain fundamental for identifying those LC patients most likely to benefit from GGT. The results from this study and previous reports suggest it is reasonable for clinicians to now consider GGT for all patients diagnosed with LC.

However, there are well-defined management recommendations to prevent and diagnose early cancers associated with PGVs, regardless of whether they are identified in patients with LC or non-LC. Here, to our knowledge, we report the PGV-including DNA damage repair (DDR) gene rate in this largest series of patients with LC and the potential clinical implications of those PGVs.

## METHODS

### Study Population

Between March 2015 and February 2022, consecutive unrelated patients with LC personal histories (PHs) underwent germline genetic testing (GGT). Eligibility was limited to those with cancer-gene panel requisition forms that included an LC diagnosis in free text and/or diagnosis code C34 from the *International Classification of Diseases, Version 10* (2022 ICD-10 CM). Patients with a reported PH of neuroendocrine tumors, nonmalignant lung conditions, sarcomas, or lung metastases were excluded. Primary non–small-cell LC (NSCLC) and small-cell LC were not analyzed separately. Review and analysis of deidentified data were approved by the Western Copernicus Group (WC) Institutional Review Board (IRB; study ID CR-001-02). The submission involved only retrospective analysis of deidentified patient information, for which waiver of signed consent was approved by the WCG IRB, protocol No. 1167406. A previously published control cohort of 10,478 individuals (average age 49.5 years, 59% female) without cancer who underwent GGT was used as a comparison group of PGV frequencies in *BRCA2*, *CHEK2*, *ATM*, and *BRCA1*, and the DNA mismatch repair (MMR; Lynch syndrome–associated) genes *MLH1*, *MSH2*, *MSH6*, *PMS2*, and *EPCAM*.^17^

### Genetic Testing

Genomic DNA was analyzed using next-generation sequencing as previously described.^18^ Briefly, requisitioned genes were fully sequenced, including exons, the 10-20 flanking intronic bases, and certain noncoding regions of interest (average 350×, minimum 50×). Single-nucleotide variants, insertions/deletions, structural variants, and intragenic copy-number variants were identified via a custom bioinformatics pipeline^19,20^ and categorized using Sherloc, a refinement of the guidelines from the American College of Medical Genetics and Genomics and the Association for Molecular Pathology.^21,22^ GGT results categorized patients as positive if they were heterozygous for a P or LP variant associated with an autosomal dominant hereditary cancer syndrome; otherwise, the results were characterized as carrier, negative, or of uncertain significance (Data Supplement, Methods).

### Genomic Ancestry

Patients' genomic ancestry was calculated using ancestry informative markers (AIMs) with reference to five continental ancestries; African (AFR), Ad Mixed American (AMR), East Asian (EAS), South Asian (SAS), and European (EUR; Data Supplement, Methods).

### Statistical Analysis

The proportions of patients classified as positive, carrier, uncertain, or negative were calculated based on the number of patients for whom the panel included the gene, and further evaluated based on genomic ancestry, PH of other cancers, and FHs of other cancers. A two-sample test for equality of proportions without continuity correction was conducted to compare the PGV rate of the overall cohort with those with only a PH of LC. Differences in PGV rates by gene between the study cohort and control cohort were assessed by χ^2^ analysis. Possible mosaic PGVs were not included in this analysis.

### Clinical Actionability

Clinical actionability of test results among positive patients was assessed based on two analyses (Data Supplement, Table S1). The first analysis was the proportion of DDR genes identified (including homologous recombination repair and MMR genes), indicating clinical trial eligibility. The second analysis was the proportion of PGVs in genes with clinical management implications for non-LC cancer types, per recommended guideline management measures and/or potential clinical trial eligibility.

## RESULTS

### Study Cohort Characteristics

A total of 7,874 unrelated patients with LC were referred for GGT. Eighty-six patients were excluded from this analysis because their requisitions indicated they had sarcoma, neuroendocrine tumors, or carcinoids. Among the remaining 7,788 patients, 71.1% were female and the mean age at the time of testing was 63 ± 15.2 years (Table 1). Genomic ancestry is displayed in Table 1 (self-reported ancestry; Data Supplement, Table S5). Patients were tested for up to 159 genes; two-thirds were tested via an 80-84 gene panel (n = 3,438, 44.1%)^23^ or a 42-47 gene panel (n = 1,745, 22.4%).^24^ The majority (77.1%) reported a PH of another cancer type in addition to their LC, most commonly breast (33.5%), gastrointestinal (23%), and genitourinary (15.4%) cancers.

**TABLE 1.**
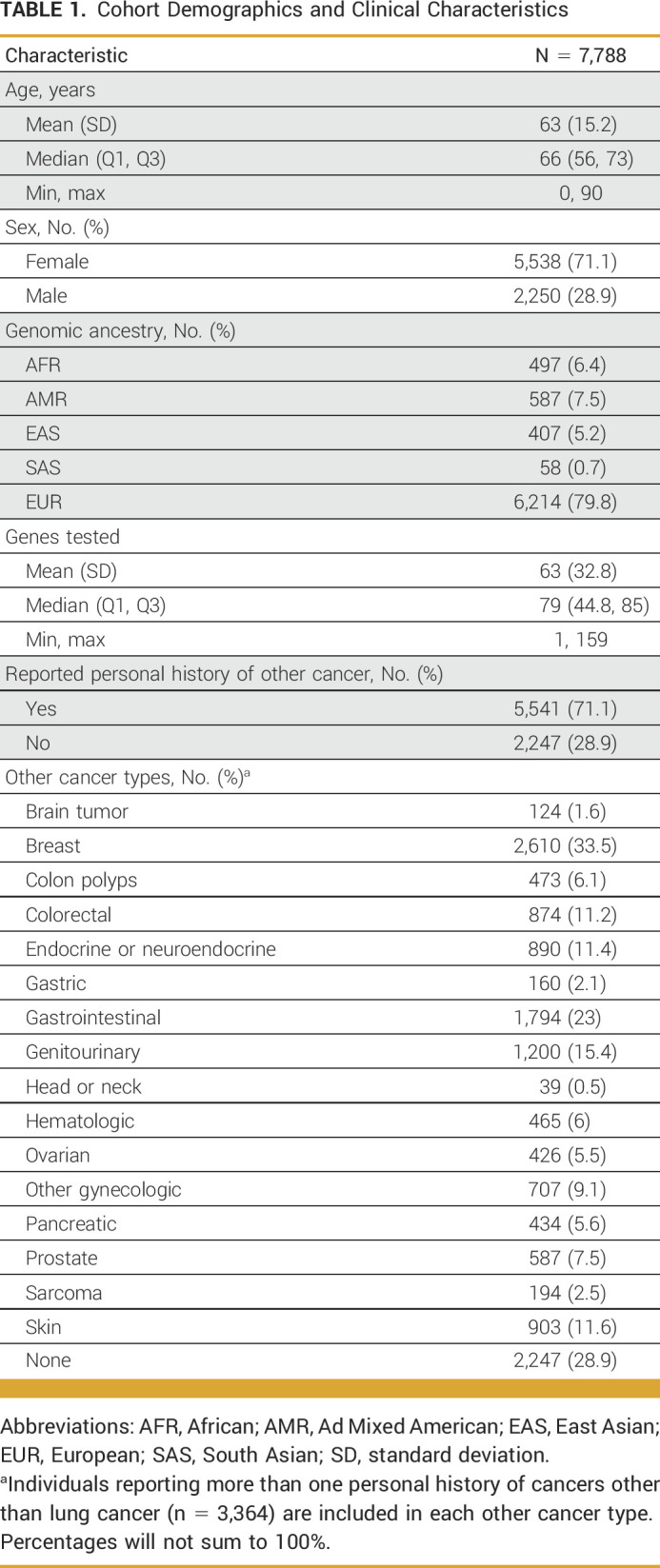
Cohort Demographics and Clinical Characteristics

### GGT Results in the Overall Study Cohort

**FIG 1.**
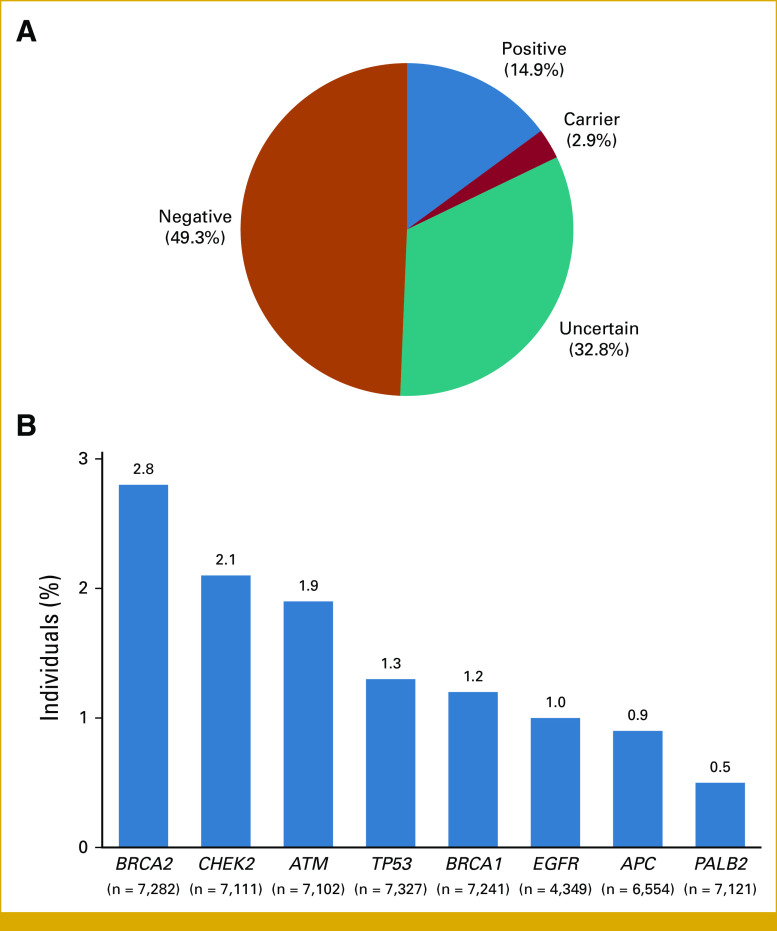
Genetic test results among all individuals in the study cohort. (A) Overall genetic test results. See the Data Supplement (Methods) (Genetic testing) for definitions of positive, carrier, uncertain, and negative. (B) Proportion of individuals with a positive result in genes. Proportion is based on the total number of individuals who had the gene ordered by their clinician. Positive rates for all genes can be found in the Data Supplement (Table S2).

A positive result was identified in 1,161 patients (14.9%; Fig 1A), a carrier result in 229 patients (2.9%), and 2,555 patients had an uncertain result (32.8%). The remaining 3,843 patients (49.3%) had no PGVs or variants of uncertain significance (VUS). Among genes evaluated in more than 1,000 patients, positive results were most common in *BRCA2* (2.8%, n = 202/7,282), *CHEK2* (2.1%, n = 151/7,111), and *ATM* (1.9%, n = 136/7,102; Fig 1B and Data Supplement, Table S2). The frequency of PGVs in *BRCA2*, *ATM*, *CHEK2*, and *BRCA1*, and MMR (*MLH1*, *MSH2*, *MSH6*, *PMS2*, or *EPCAM*) among these patients with LC was significantly higher than the frequencies seen in the control cohort of unaffected individuals undergoing proactive genetic screening (*P* < 10^−5^; Table 2). Positive results in *EGFR* (0.9%, n = 41/4,349) were also observed. The proportion of patients with a positive result across all genes is listed in the Data Supplement (Table S2). A subset of patients with positive results (n = 67, 5.8% of positives) were found to have possibly mosaic PGV (Data Supplement, Table S2).

**TABLE 2.**
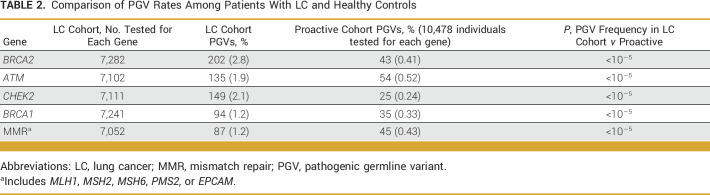
Comparison of PGV Rates Among Patients With LC and Healthy Controls

**FIG 2.**
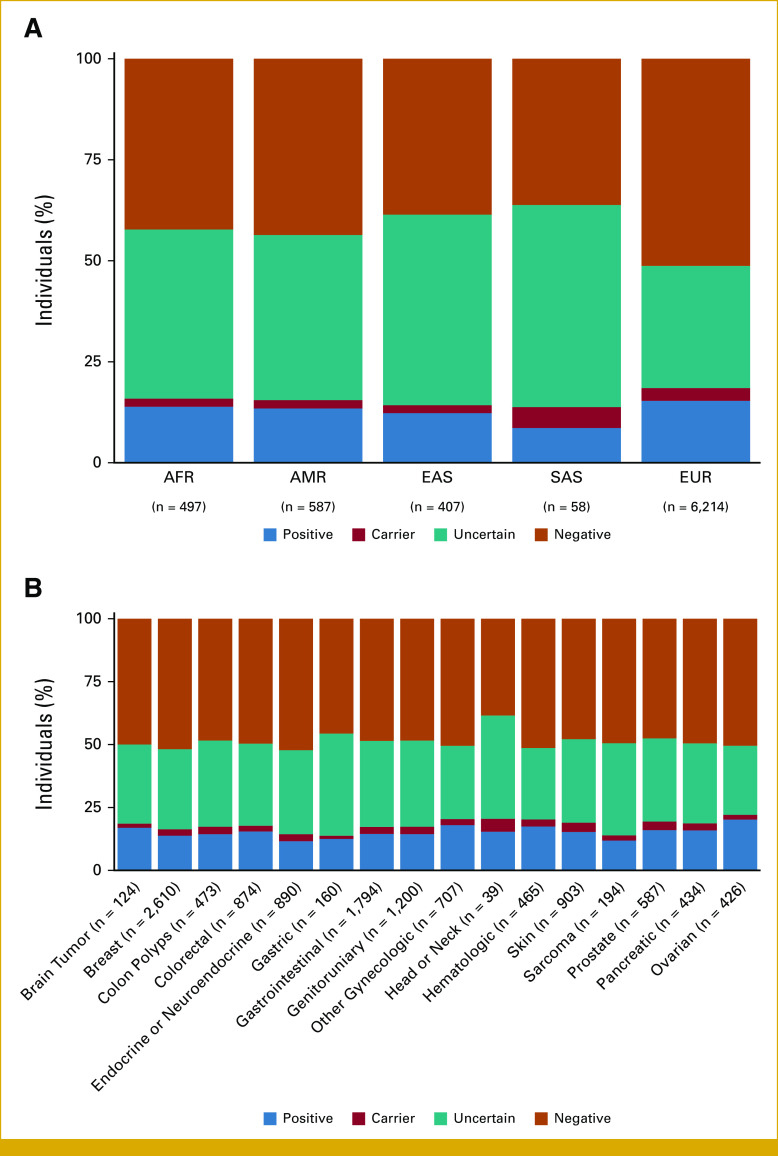
Genetic test results by (A) genomic ancestry and (B) personal history of other cancers. Proportion is based on the number of patients in each category, with the total number of patients in each category reported on the *x*-axis. Of note, 20 patients (0.02% of cohort) did not demonstrate a clearly predominant genomic ancestry and are not included in the analysis in panel (A). AFR, African; AMR, Ad Mixed American; EAS, East Asian; EUR, European; SAS, South Asian.

The positive rate varied based on genomic ancestry and PH of other cancer types. Patients with EUR genomic ancestry had the highest positive rate (18.5%), followed by AFR (15.8%), AMR (15.5%), EAS (14%), and SAS (13.7%) ancestries (Fig 2A). The VUS rate was significantly higher for patients with AFR, AMR, EAS, and SAS genomic ancestry (40%-50%) compared with those with EUR genomic ancestry (30%; *P* < .00001). The positive rate was highest among patients with a PH that included ovarian (20.2%) and other gynecologic cancers (18%; Fig 2B).

Among the 1,161 positive patients, 61.3% (n = 712) were potentially eligible for a clinical trial on the basis of an identified PGV in a DDR gene (Data Supplement, Table S1). Nearly all of the 1,161 patients with a PGV in a DDR gene (n = 1,104, 95.1%) had findings in a gene with clinical management implications for other cancer types, including clinical management recommendations on the basis of professional guidelines or potential eligibility for clinical trials.

### Findings for Patients With LC as Their Only Cancer

**FIG 3.**
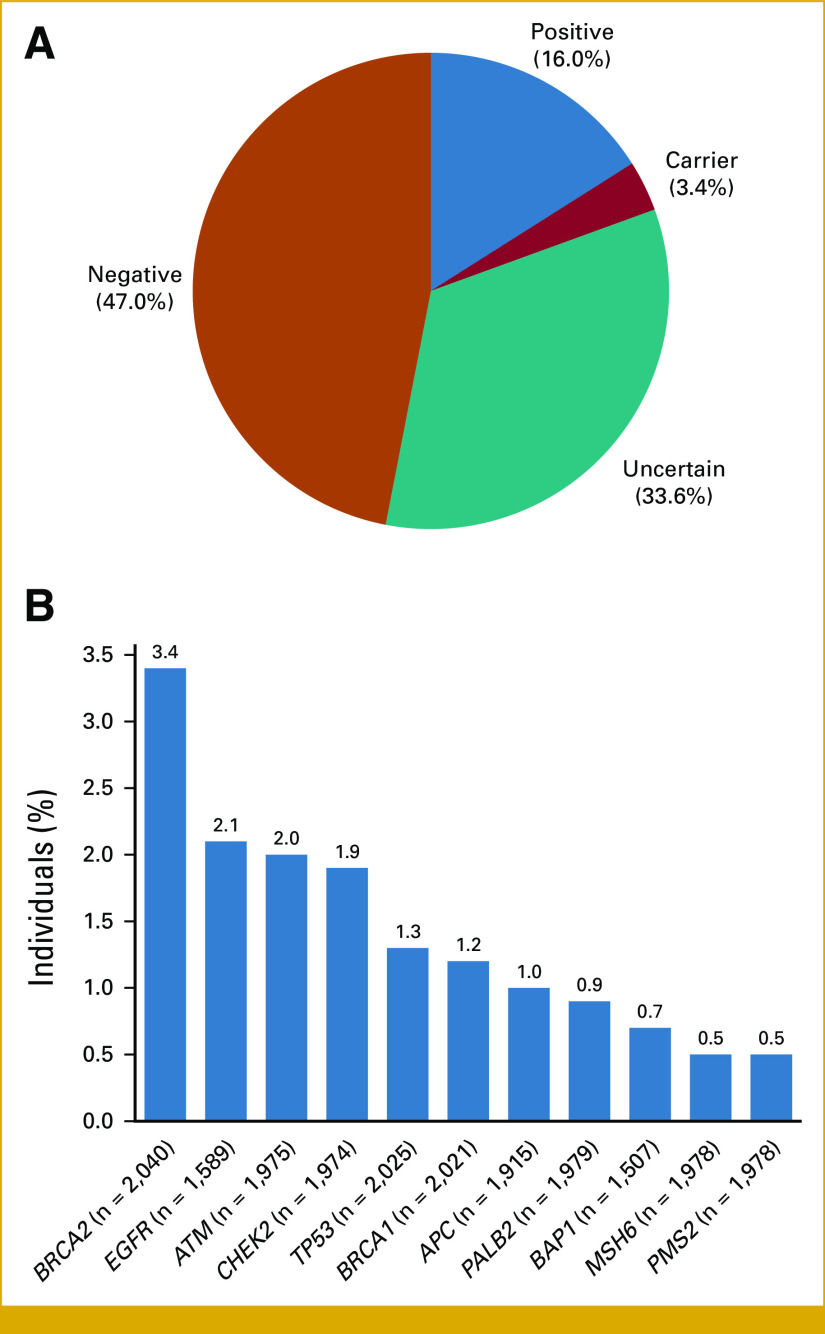
Genetic test results among patients with only a personal history of lung cancer, per clinician report. (A) Overall genetic test results. See the Data Supplement (Methods) (Genetic testing) for definitions of positive, carrier, uncertain, and negative. (B) Proportion of patients with a positive result in genes. Proportion is based on the total number of patients who had the gene ordered by their clinician. Positive rates for all genes can be found in the Data Supplement (Table S3).

The majority of patients in the cohort had a PH of at least one other cancer (n = 5,541, 71.1%; Table 1). To assess whether the findings of the overall cohort were biased because of a PH of other cancer types, the remaining 2,247 patients with primary LC and no PH of other cancer types were analyzed separately. The mean age at the time of testing for these patients was 58.7 ± 16.8 years. In this cohort, there were 360 (16%) positive patients, 77 (3.4%) carrier patients, and 755 (33.6%) who received an uncertain result (Fig 3A). The positive rate was not significantly different compared with patients with a reported PH of another cancer (n = 801/5,541, 14.5%; *P* = .079).

The most common positive results in this cohort of patients with only a PH of LC were in *BRCA2* (3.4%, n = 70/2,040), *EGFR* (2.1%, n = 34/1,589), and *ATM* (2%, n = 40/1,975; Fig 3B, Data Supplement, Table S3). Similar to the overall cohort, 230 (63.9%) may have been eligible for a clinical trial because the identified PGV was in a DDR gene and 352 patients (97.8%) had a positive PGV that has clinical management implications for other cancer types.

### Findings for Patients With LC but Without PH or FH of Other Cancers

Similarly, 14.3% (n = 83/582) of patients without a PH of other cancers *and* without reported FH of cancer received a positive result (Data Supplement, Fig S1 and Table S4). Potential eligibility for tumor-agnostic clinical trials because of a PGV in a DDR gene was found in 62.7% of these patients, and 94% of patients had clinical management implications, such as surveillance recommendations because of an elevated risk for other cancer types and genetic counseling of relatives. Furthermore, results were similar regardless of whether patients were stratified according to both PH of other cancers and a FH of cancer (Data Supplement, Fig S2).

### Clinical Characterization of Patients With a Positive Finding in *EGFR*

Forty-one patients had the p.T790M PGV in *EGFR*. Before germline testing, *EGFR* p.T790M was reported on somatic testing in all patients (n = 33) who reported somatic testing results. Among these 41 patients, the mean age at LC diagnosis was 57.2 ± 11.5 years. The cohort was predominantly female (n = 31, 75.6%) and EUR (n = 32, 78%). The remainder of the cohort had AFR ancestry (n = 8, 19.5%) or multiple ethnicities (n = 1, 2%). Of the 18 patients with information related to smoking history, 15 (83.3%) were nonsmokers. Ten other cancers were reported in seven patients (17.1%), including breast (n = 4), colorectal (n = 1), endocrine or neuroendocrine (n = 1), uterine (n = 1), ovarian (n = 1), pancreatic (n = 1), and kidney (n = 1). Among 36 patients with family histories available, 29 (80.6%) reported a first- or second-degree relative with LC.

## DISCUSSION

In this study, approximately one in seven patients with LC (14.9%) who underwent GGT had a PGV. Although this frequency is higher than previously reported, other studies involved different methodologies and different patient populations, making cross-study comparisons problematic. For example, in a study reported by Mukherjee et al, germline PGV in high-/moderate-penetrance genes were demonstrated in 222/5,118 patients (4.3%) with metastatic NSCLC. However, in that study, only patients with paired tumor-normal tissue were included and 30% of patients had a FH of any cancer, a PH of multiple tumors, or an early age of LC diagnosis, whereas up to 71.1% of patients on this study had a reported PH of another cancer, and advanced stage of their LCs was not required. The frequency of each of those PGVs identified was also less in the study by Mukherjee et al^25^: *BRCA2* (1.1%), *CHEK2* (0.58%), and *ATM* (0.51%). Parry et al reported that among 555 patients with lung adenocarcinomas, five known PGVs were identified in 2.5% of their patients. They concluded that for a subset of patients with lung adenocarcinoma, at least 2.5% to 4.5% carry germline variants that have been linked to cancer risk.^26^ From a study of unselected patients with advanced cancers, 1.4% (41% with LC) were found to have suspected or putative germline finding on tumor testing.^5^ From a study of 1,200 Chinese patients with NSCLC, 2.2% harbored inherited germline mutations, with EGFR being the most common PGV identified (1.05%).^7^ Taken together, it seems reasonable to conclude that PGVs are infrequently identified in patients with LC but are seen in nearly 15% of patients with LC when the basis for ordering GGT is ostensibly the diagnosis of LC, as was the inclusion criteria for this study. Irrespective of the reason for the higher frequency of PGVs identified in this study, in comparison with other studies, this real-world finding suggests that far more patients diagnosed with LC and their families might benefit from GGT than are currently offered testing.

The current most common reason that GGT is recommended in any patient with cancer is a PH and/or FH suggesting a high pretest probability of uncovering a PGV. However, the recent Cancer Moonshot 2.0 Initiative includes recommendations that all patients diagnosed with cancer be assessed to determine eligibility for genetic testing, not only those patients with strong PH/FHs, in part because of socioeconomic disparities between patients referred for testing, but also because studies suggest reported FHs are not reliable for estimating pretest probabilities.^27,28^ Cancer Moonshot 2.0 also recommends that payers not deny visits for genetic counseling on the basis of the pretest probability of patients carrying a PGV. Although the cost of universal testing of patients with cancer would be substantial, studies have shown that universal testing for the most common PGVs is cost-effective and saves lives, in part related to measures for early diagnosis and prevention endorsed by the National Comprehensive Cancer Network.^26^ Currently, among patients not meeting criteria for GGT, but who still wish to undergo GGT, only those patients who can afford to pay out of pocket for GGT will be tested. Thus, almost paradoxically, it is anticipated that if the universal referral recommendation by Cancer Moonshot 2.0 is adopted, since most patients do not meet current National Comprehensive Cancer Network Guidelines than do, the gaps between socioeconomic groups in whom is tested will likely be widened.^29^

Here, 14.3% who reported no FH of cancer had a positive result, suggesting that restricting testing to patients with LC with a FH of cancer may limit access for a number of patients and their families to the benefits of this genetic information. Several large studies show that a cancer diagnosis alone is a biomarker that predicts PGVs, one demonstrating over half of the clinically actionable PGVs would have been missed using guideline-directed testing on the basis of FH.^13,30^

When an incidental PGV finding is returned after somatic testing or when treatment decisions are informed by identifying a PGV, GGT is also National Comprehensive Cancer Network–recommended. The only example of referral for GGT based solely on a LC diagnosis is when tumor sequencing identifies an *EGFR* p.T790M in the absence of previous *EGFR* tyrosine kinase inhibitor therapy.^31^ In our study, 0.9% had *EGFR* p.T790M PGVs. Patients confirmed to have *EGFR* p.T790M PGVs appear to have roughly a 23% increased risk of LC, and screening for LC might be considered in these patients and for other cancers if proven to be *EGFR*-associated.

Our results support the emerging evidence that PGVs in *TP53* and *EGFR* T790M are not the only LC-predisposing PGVs. There were statistically significant increased frequencies of PGVs in this study in *BRCA2*, *ATM*, *CHEK2*, *BRCA1*, and Lynch syndrome–associated MMR genes compared with the frequencies seen in the referenced control cohort of unaffected individuals undergoing proactive genetic screening (*P* < 10^−5^) of 6.8-, 3.6-, 8.8-, 3.6-, and 2.7-fold, respectively. These findings represent further evidence suggesting that, like *EGFR* p. T790M, these PGVs are cancer-predisposing. However, these PGVs should not be considered causative of LC. If these PGVs are established as LC-predisposing, additional studies will be needed to more precisely estimate the penetrance of these PGVs related to LC, particularly in the settings of other LC risk factors, such as cigarette smoking or harboring polymorphisms that affect tobacco metabolism.^32^

*BRCA2* PGVs were the most common PGVs identified, representing 17.4% of patients with positive findings, and numerous reports have suggested that *BRCA2* PGVs, like *EGFR* T790M, are LC-predisposing.^4,33-41^ Although a PGV in *BRCA2* in a patient with LC is not a US Food and Drug Administration–approved indication for poly (ADP-ribose) polymerase (PARP) inhibitor use, such use could be considered if standard therapies have been exhausted or as part of a tumor-type agnostic PARP inhibitor clinical trial (eg, NCT02401347, NCT03344965).^42^ *CHEK2*, a DDR gene also involved in the activation of *TP53*, has previously been implicated as possibly LC-predisposing,^33^ and increased screening for breast, colorectal, prostate, and other cancers is indicated when a PGV in *CHEK2* is identified.^43,44^ It has also been suggested that PGVs in *CHEK2* may open PARP inhibitors as a potential treatment option and may predict resistance to anthracycline therapy.^45,46^ A probable association between LC and *ATM* has been previously established, with case-control studies estimating odds ratios ranging from 3.66 to 4.6.^47,48^ In addition to these PGVs being potentially LC-predisposing, patients and their family members can benefit enormously from recommended measures to prevent and diagnose early cancers for which these PGVs confer increased risk.

The majority of uncovered PGVs were in DDR genes and these warrant special attention because these PGVs inform targeted therapies for associated cancers and clinical trial eligibility. For example, identification of PGVs in DDR genes associated with non-LC informs treatment options with PARP inhibitors, improves outcomes, and, in some cases, increases overall survival for patients with breast, ovarian, pancreatic, and prostate cancer.^44,49^ Investigators recently reported that patients with small-cell LC who carry PGVs in *BRIP1*, a DDR gene, appear to have more benefit from platinum-based therapy than those who lack the same *BRIP1* PGV.^50^ Overall, the potential treatment benefit for patients with LC with DDR PGVs remains theoretical and deserves further investigation.

PGV rate in this study varied with genomic ancestry, calculated using AIMs embedded in the clinical genetic testing panel, with the highest percentage of findings in patients with EUR genomic ancestry. Using genomic ancestry enabled more inclusive analysis, as advocated by Oni-Orisan et al,^51^ and is key as research suggests implications of self-reported ancestry for patients with NSCLC.^52^ Genomic ancestry stratification of our cohort revealed a statistically disproportionate number of VUS among patients of historically under-represented populations, compared with those of EUR ancestry. This reinforces the need for future studies on PGVs and patient outcomes, stratified by genomic ancestry, to understand their impact on LC. It also underscores the need for equitable access to genetic testing for patients across ancestries.

Aspects of this retrospective study warrant comment. First, limited or no clinical data were available for assessing which patients in this cohort would have met National Comprehensive Cancer Network Guidelines for non-LC cancer types that would have resulted in referral for GGT. In particular, it is possible that GGT for many patients in this study was ordered because of a PH of another cancer type (ie, up to 71.1% of the cohort) or because of a FH of other cancer types. Although it would seem likely that many of the patients with multiple cancers would have been eligible for referral for genetic counseling, we do not have data to suggest what proportion of this group were seen by genetics, and of those, for what proportion GGT was recommended. Notably, we found the frequency of PGVs was not different between patients with only a LC history, patients with LC and PHs of non-LC, and patients with LC and PHs of non-LC and FHs of cancer. The finding that most patients had a PH of other cancers suggests that GGT after those earlier cancers were diagnosed may have allowed for increased screening and prevented other primary cancers from developing later.^53^

Although the patient population may be representative of the average LC cohorts who undergo GGT testing, our cohort does not appear to be representative of the average LC cohort. For example, 71% of the patients tested were female, which does not correlate with the sex-stratified incidence of LC and may be related to reported FH of *BRCA1/2*-related cancers in this group contributing to GGT. The diagnosis of LC was required for study eligibility, but we cannot be certain that all patients had, in fact, a diagnosis of primary LC. Although it is unclear what indication beyond LC (if any), in those patients with only a PH of LC, moved clinicians to order GGT, this study was not designed to assess differences in clinical practice. It is certainly possible that providers did not report the true indication they had used for GGT testing in this cohort. As with other studies of this type, we relied on the integrity and thoroughness of the information the ordering clinician provided on the test requisition. However, it is unlikely that providers would fail to report the true reason for ordering GGT testing, particularly given the lack of coverage for GGT in patients with solely a LC diagnosis. Ultimately, we are unable to verify the primary or secondary nature of the reported diagnosis, PH, and FH, because of the real-world setting of this study.

Another limitation is that included patients were referred for testing with various panel sizes (including single gene testing), and so, the PGV rate may be an underestimate, compared with if all patients had received 84-gene panel testing. Of note, only 4,349 patients were tested for *EGFR*, whereas the other genes with the highest PGV rates had more than 6,500 patients tested.

Finally, 5.8% of positive patients had possibly mosaic positive results on a platform optimized to identify germline variants. Such a finding could represent germline mosaicism, clonal hematopoiesis of indeterminate potential, or circulating tumor cells. Additional hematologic evaluations or GGT of other tissues (eg, fibroblasts) could help to determine the clinical significance of these results.

To our knowledge, this is the largest study using clinical genetic testing to characterize both oncogene and tumor suppressor gene PGVs in a population of patients with LC stratified by genomic ancestry. It is estimated that there are currently 576,924 Americans living with a diagnosis of LC, roughly 85,962 of whom could have a PGV on the basis of our findings.^54^ These results indicate that certain PGVs are particularly prevalent in patients with LC, suggesting that additional studies are needed to confirm which PGVs are LC-predisposing. Also, we found the frequency of PGVs and VUS vary according to genomic ancestry. Taken together, these findings suggest that once the patient has been informed of the risks, benefits, and uncertainties related to testing, it may be of interest to routinely examine all patients with LC for the presence of PGVs in cancer-risk genes, benefiting patients and their relatives.
